# The effectiveness of body age-based intervention in workplace health promotion: Results of a cohort study on 9851 Danish employees

**DOI:** 10.1371/journal.pone.0239337

**Published:** 2020-09-17

**Authors:** Karina L. S. Husted, Sune Dandanell, Janne Petersen, Flemming Dela, Jørn W. Helge

**Affiliations:** 1 Department of Biomedical Sciences, Faculty of Health Science, University of Copenhagen, Copenhagen, Denmark; 2 Department of Physiotherapy and Occupational Therapy, University College Copenhagen, Copenhagen, Denmark; 3 Department of Public Health, University of Copenhagen, Copenhagen, Denmark; 4 Center for Clinical Research and Prevention, Bispebjerg and Frederiksberg Hospital, Copenhagen, Denmark; 5 Department of Geriatrics, Bispebjerg Hospital, Copenhagen, Denmark; Universitat de les Illes Balears, SPAIN

## Abstract

**Introduction:**

The aging population emphasize the need for effective health promotion interventions. The workplace is a prioritized setting for health promotion to reach widely within a population. Body age can be used as a health-risk estimate and as a motivational tool to change health behavior. In this study we investigate body age-based intervention including motivational interview and its effect on health, when applied to real life workplace health promotion.

**Material and methods:**

Body age-based intervention was performed in 90 companies on 9851 Danish employees from 2011–2017. Metabolic risk factors were assessed, body age score was determined and an individualized motivational interview was conducted at baseline and follow-up. Change in body age score, single risk factors, smoking habits and metabolic syndrome were analyzed. The body age score is a composite score comprising 11 weighted variables. A body age score ≤ 0 is preferred, as this elicit a younger/healthier or equal body age compared to chronological age.

**Results:**

At 1.3 year follow-up the unhealthiest employees were less likely to participate. Within follow-up participants (39%, n = 3843) body age had improved by a decline in mean body age score of -0.6 and -0.7 years for men and women, respectively (p<0.001). Number of employees with metabolic syndrome had decreased from 646 at baseline to 557 at follow-up (p = 0.005) and 42% of smokers had quit smoking (p<0.001).

**Conclusion:**

On the basis of this study, we suggest that body age assessment motivates to participate in workplace health promotion, affect high risk behavior such as smoking thus have potential in public health promotion.

## Introduction

The macroeconomic implications of the aging workforce depends mainly on how long people continue to work [1]. In western countries legislated statutory retirement age is set to increase in line with the increase in life expectancy [2] thus, a healthy aging workforce is increasingly important. Unfortunately, a healthy aging workforce is challenged by inactive lifestyle and increasing prevalence of obesity and non-communicable diseases (e.g. cardiovascular disease and type 2 diabetes) [3, 4]. The workplace is a favorable setting to reach widely within a population because individuals with similar profiles, (e.g. lifestyle and socio-economic status) tend to cluster at different work-sites [5, 6]. A frequently used tool in workplace health promotion is health risk assessment (HRA) [7]. HRA produce a risk profile for individuals based on demographic, behavioral and biometric information. Motivational interview (MI), a client centered technique focusing on exploring and resolving ambivalence towards behavior change, has proven effective when it comes to change of health behavior [8]. It has been suggested that adding motivational interview (MI), will increase the effectiveness of HRA in workplace health promotion [9].

Recently, HRA used to produce individual biological age (also named body age and fitness age) as a health-risk estimate and motivational tool is increasingly applied [10, 11]. Heterogeneity in functional status and vulnerability to disease can be assessed by biological age. This heterogeneity is due to non-modifiable factors such as genetics and modifiable factors such as lifestyle [12, 13]. Biological age is commonly constructed from a number of modifiable risk factors assessed as reliable biomarkers of aging and proven valid as a health-risk estimate and prediction of mortality compared to chronological age [14–16]. Being older (or younger) than ones chronological age is easily translated into risk of disease and vigor and can be a motivation for healthy lifestyle. To our knowledge, only one study has been identified evaluating the effect of using body age estimation in workplace health promotion [11]. To adequately assess the potential of body age as a tool in workplace health promotion more research is needed.

Therefore, this study aims to evaluate the effectiveness of a commercially developed body age-based intervention (BAI) including MI in a large representative sample of the Danish workforce. The objectives are change in body age score and the associated changes in health behavior and single risk factors. In addition, we will include metabolic syndrome as an effect measure related to risk of future non-communicable diseases.

## Material and methods

### Study design

This is a retrospective database cohort study carried out by a Danish private health-care company in 90 Danish companies from January 2011 to February 2017. The companies were recruited from the Danish private health-care company’s list of previous costumers. The 90 companies were primarily private representing 97% (41% Financial, 32% Energy, 5% Transport, 5% Consultancy, 2% Food Sector Industry and 12% other), and public companies representing 3%.

The Danish private health-care company is the founder and provider of the BAI. The company had previously used the body age estimate developed by Polar (Polar Electro Inc., Kempele, Finland) [17]. Because the use of risk scores based on foreign population have been shown to produce poor results [18] the Danish company developed their own body age estimate on the basis of data from previous health screenings of 10.000 Danish employees. The BAI is designed to measure changes in variables associated with aging due to behavior and lifestyle.

### The intervention

The BAI consisted of an individual 60–70 minutes session including a health screening, body age estimation and MI (comprising ≈20 minutes). The BAI was conducted at the workplace within working hours. The health screening included assessment of metabolic function (fasting blood glucose and cholesterol profile), cardiorespiratory function (blood pressure and maximal oxygen consumption (fitness level)), body composition (weight, height, waist circumference, fat percentage and fat-free mass) and physical performance (handgrip strength, upper arm strength, thigh strength and flexibility of the hamstrings). Immediately after the health screening a standardized report was given to the employees containing their body age. Employees where informed on how the test results had influenced their body age result. This report formed the basis for the MI.

The Danish private healthcare company visited the companies twice, providing the employees with the opportunity of a baseline and follow-up test. No other intervention beside the BAI including MI was provided. The Danish private health care company aimed to do follow-up tests within 1 year, however, this was essentially decided by the employers of the companies. The test was carried out by health care professionals (sports physiologists and physiotherapists) educated, trained and supervised in the body age protocol and in techniques of MI [19]. Participation was voluntary for the employees and financed by the employers, without incentives to participate or criteria’s for participation.

### Study population

**Fig 1:**
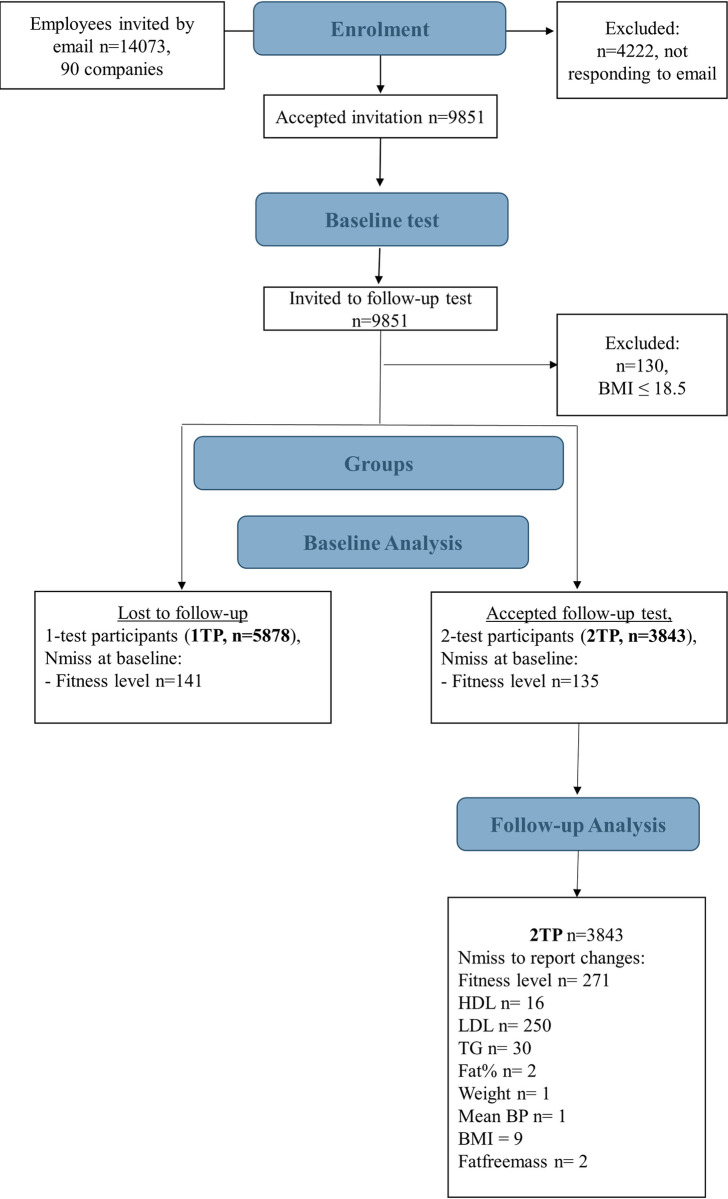
Flow chart of study population. *Nmiss* number of missing variables, *HDL* High density lipoprotein, *LDL* Low density lipoprotein, *TG* Triglycerides, *FAT%* body fat percent, *MeanBP* mean blood pressure, *BMI* Body mass index.

In total 14073 employees received an invitation by email, and 9851 (70%) chose to participate. Of the 9851 employees the study population consist of 5878 (60%) employees who participated at baseline only (from here on referred to as 1 test participants (**1TP**) and 3843 (39%) who also participated at follow up (from here on referred to as 2 test participants (**2TP**). As this study investigated the beneficial effects on obesogenic related lifestyle, employees with a low BMI (≤ 18.4) at baseline were excluded from the study population (n = 130 (1%)) (Fig 1).

### Ethical approval

This study was approved by the University of Copenhagen Research Ethics Committee for Science and Health (504–0056/19–5000) and by the Data Protection Agency (SUND–2018–17). Data was retrieved from the database of the Danish private healthcare company fully anonymized, why consent was not collected from participants. Data is reported in accordance to the STROBE statement and follows the checklist included in reports of cohort studies [20].

### Measurements and procedures

Employees were tested individually and were requested to fast minimum 3 hours before the test. Additionally, to standardize hydration level, employees where ask to drink 0.5 L of water 2 hours prior to the test. Blood from a finger prick test was used to measure glucose concentration (Accu-Check Aviva meter, Indianapolis, Indiana, USA), total cholesterol (TC), high-density lipoproteins (HDL), low-density lipoprotein (LDL) and triglycerides (TG) (Alere Cholestech LDX analyzer, Hayward, USA). With an alcohol swab the center of a finger was cleaned. When dry, a single-use lancet pricked the selected site. The finger was gently squeezed and the first drop of blood was wiped away as it may contain tissue fluid. Again, the finger is squeezed gently and 30μl is collected in a capillary tube, taking caution that the blood drop does not touch the skin. The capillary tube must be filled within 10 seconds. Secondly, a new blood drop is applied on the blood glucose meter test strip, and inserted in the meter. Blood pressure was measured at rest in the supine position using an automatic monitor (Boso-medicus control, Jungingen, Germany) and body composition was measured by bio impedance (TANITA -SC330 S, Tokyo, Japan). Waist circumference was measured between the 12^th^ costae and crista iliaca. Fitness level was assessed using a two-point submaximal cycle test ad modum Ekblom-Bak [21] on an electromagnetically braked cycle ergometer (Monark 828E, Vansbro, Sweden). Heart rate and workload was recorded at steady state after 6 and 10 min, respectively. Maximal workload (W_max_) was extrapolated on the basis of theoretical maximal heart rate (220-age) and ${\dot{V}O}_{2\;\mathrm{peak}}$ calculated based on the assumption of a cycling efficiency of 23%, an energy-oxygen equivalent of 21.1 kJ/L O_2_ and a basal metabolic rate of 0.25 L O_2_/min. Grip strength was measured with handheld dynamometer (Jamar J00105, Lafayette, USA) in a standing position with the arm by the side. Three attempts were allowed and the highest result registered. Leg endurance strength was assessed by wall-sit hold, performed with the back against a wall, and a 90˚ flexion of the hip and knee joint. Upper body strength was assessed via the maximal number of pushups. A valid pushup was defined by the chest touching a foam roller on the floor—men being on their toes, women being on their knees as starting position. A sit-and-reach test was used to assess flexibility. The participants sat on the floor with 90˚ flexion of the hips and with a straight back. The sit-and-reach bench (ACUFLEX I, Rockton, USA) was pushed against the feet and the participant reached forward pushing the reach indicator away, in a fluent movement. Finally, participants were asked about number of cigarettes smoked per day.

### Determination of body age

Body age is the sum of chronological age and the body age score:

Bodyage=Chronologicalage+BAscore.

The body age score is a composite score comprising 11 weighted variables assessed in the health screening. The variables included are: fitness level, fat%, total cholesterol, blood glucose concentration, mean blood pressure, waist circumference, handgrip strength, push up, wall sit, sit and reach, and smoking habits. A body age score ≤ 0 is preferred, as this elicit a younger/healthier or equal body age compared to chronological age. The body age score was determined using the following algorithm:

$$BAscore=\sum_{i=1}^{N}\Delta_{i}^{V}\times W_{i}^{V}+\Delta^{BG}+\Delta^{SH}$$

Where $\Delta_{i}^{V}$ is the age value given for each of the 9 variables (not including blood glucose concentration and smoking habits), the $W_{i}^{V}$ is the corresponding weight. The age value depends on how the corresponding test results varies in statistical data of age and sex-related peers. National recommendations of variables relationship with health and risk of disease was used to determine the weighting (Table 1).

**Table 1:** Weighting of variables.

Variable	$W_{i}^{V}$^a^
Fitness level	31.1%
Fat percent	17.8%
Total cholesterol	13.4%
Mean blood pressure	13.4%
Waist circumference	6.7%
Handgrip strength	4.4%
Push up	4.4%
Wall sit	4.4%
Sit and reach	4.4%
*TOTAL*	100%

The weight of each variable relative to its estimated importance of health and risk of disease ^a^$W_{i}^{V}$ = the weight in percent assigned to each variable.

*N* is the total number of variables applied in the algorithm (11 variables) and *i* indicate the specific variable (e.g. fitness level). Parameters Δ^*BG*^ and Δ^*SH*^ represent the age value given based on blood glucose (BG) concentration and smoking habits (SH). A blood glucose concentration >6.1 mmol/L results in an age value of +4 years, and smoking attributes an age value in accordance to the number of cigarettes smoked per day (CPD): 1–10 CPD attributes +4 years, >10 CPD attributes +8 years and >15 CPD attributes +10 years. Summation of the 11 age values produce the overall body age score.

### Statistics and data analysis

A merged dataset from the 6-year data collection was extracted by the IT department of the Danish private healthcare company and cleaned before analysis. Normal distribution was checked using q-q plots and histograms, variance homogeneity was checked by plotting residuals versus predicted values. Descriptive statistics at baseline were presented as medians with IQR. Baseline comparison of 1TP and 2TP was analyzed using linear regression for continuous variables and logistic regression for categorical variables. Changes in outcomes between baseline and follow-up were compared using the *proc mixed* procedure of each outcome on follow-up adjusted for age at baseline and follow-up time. Thus, interpretation of results are how much more the outcome has changed per year beside the average age development. Residuals for the analysis where checked for normal distribution to ensure that the underlying assumptions of the statistical model were met. When normal distribution did not fit the model log transformation was used successfully. Linearity of covariates were checked by visual inspection of residuals plots against covariates, if linearity assumption was not met, covariates was modelled using splines. McNemar’s test was used to analyze changes in smoking habits and Chi-square test was used to test for independency of sex. All statistical analyses were done in SAS Enterprise Guide 7.1. Statistical significance was considered at *p<0*.*05* in all comparisons. Metabolic syndrome was assessed using the definition set out by the International Diabetes Federation [22].

## Results

Based on the email invitation 70% (n = 9851) accepted and participated in the BAI at baseline. The study population was 41.3 years on average (range 18–70 years) and 63% where men. Overall 57% were normal weight (BMI 18.5–24.9) and 9% were smokers.

### 1TP versus 2TP baseline characteristics

**Fig 2:**
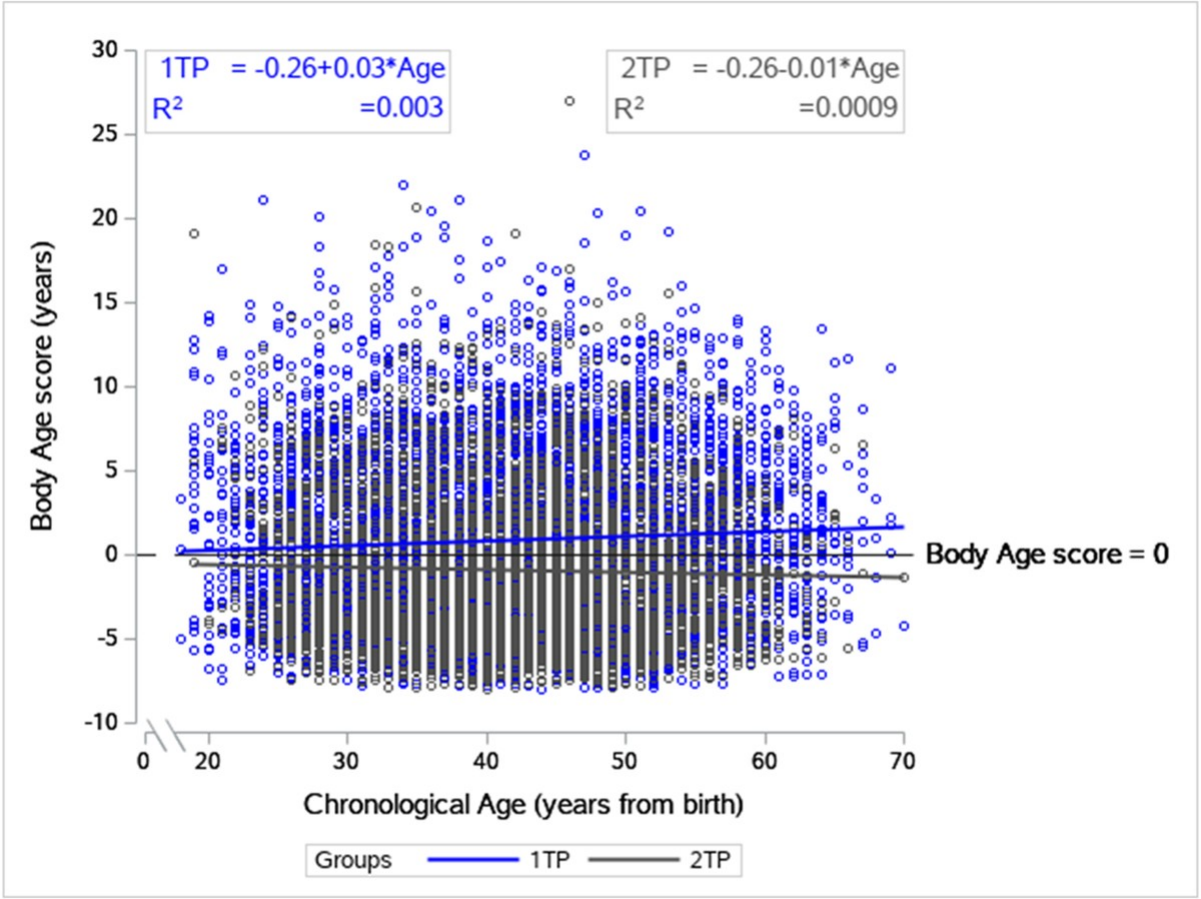
Fitted regression of body age score as a function of chronological age at baseline. The figure show that there is an association between having a positive body age score, and thereby a higher risk, and not participating at follow-up. The blue line represents body age score for 1 test participants (1TP) and the grey line represents body age score for 2 test participants (2TP). The black line is a reference line for body age score of zero that is, no difference between chronological and body age.

Table 2 shows the baseline characteristics of the 11 variables included in the body age model by groups and Fig 2 shows the related body age scores as a function of age.

**Table 2:** Baseline characteristics.

	Groups		*P*^a^	*P*^b^ adjusted
	1TP	2TP		
	Median (IQR)	Median (IQR)		
Women, n *(%)*	2182 (37.1)	1430 (37.2)	0.93	0.89
Chronological age, *years*	41 (33; 49)	42 (35; 48)	0.0006	-
Body age, *years*^c^	41.3 (32.7; 50.4)*	40.8 (33.4; 48.2)*	<0.001	-
BMI, *kg/m*^2^	24.5 (22.4; 27.1)	24.2 (22.2; 26.3)	<0.001	<0.001
Current Smoker, *(%)*	631 (11%)	245 (6%)	<0.001	<0.001
Mean blood pressure, *mmHg*	105 (98.5; 113)	104 (98; 110)	<0.001	<0.001
Total Cholesterol, *mmol/L*	5.0 (4.3; 5.6)	4.9 (4.4; 5.6)	0.9	0.3
Blood glucose, *mmol/L*	5.0 (4.7; 5.4)	5.0 (4.7; 5.4)	0.4	0.2
Body Fat *%*	22.9 (17.8; 29.4)	21.9 (17.4; 28.2)	<0.001	<0.001
Waist circumference, *cm*	88 (80; 97)	87 (80; 94)	<0.001	<0.001
Fitness level, *ml/min/kg*^c^	37 (31; 44)	40 (34; 47)	<0.001	<0.001
Push Ups, *No*. *of*	25 (16; 32)	25 (18; 32)	0.02	0.0002
Wall sit, *min*	1.6 (1.1; 2.1)	1.7 (1.3; 2.2)	<0.001	<0.001
Handgrip, *kg*	47 (34; 56)	48 (35; 56)	0.04	0.03
Sit and Reach, *cm*	35 (28; 40)	34 (29; 40)	0.4	0.2

Comparison of baseline characteristics for 1-test participants (1TP, *n = 5878*) and 2-test participants (2TP, *n = 3843*). Continuous data are represented as medians with interquartile range (IQR); categorical data as absolute and relative frequencies. Body mass index (BMI), mean blood pressure, total cholesterol, blood glucose, body fat% and waist circumference were log transformed prior to analysis.

^a^P value using regression analysis and logistic regression.

^b^P value adjusted for age.

^c^Missing values were observed for fitness level and body age (due to missing fitness level data) why comparison of 1TP and 2TP is between n = 5737 and n = 3708, respectively.

*Significant different from chronological age p<0.001 (paired t-test).

Fig 2 visualize that 1TP are associated with positive body age scores and 2TP are associated with negative body age scores, indicative of an unhealthier profile for employees lost to follow-up (1TP). With increasing age this association becomes more pronounced *(p<0*.*001)*. This is reflected in a lower body age for 2TP compared to 1TP *(p<0*.*001)* (Table 2).

The majority of variables included in the body age model were significantly different between groups, except for total cholesterol and blood glucose concentration (Table 2). BMI, mean blood pressure, body fat% and waist circumference were lower for 2TP and this was concurrent with a lower prevalence of smokers. Baseline fitness level and strength related measures were higher in 2TP, with no difference in flexibility between the groups. These differences remained significant after adjusting for age (Table 2). The proportion of participants with obesity (BMI ≥30) were higher in 1TP compared to 2TP (p< 0.0001) with no differences in the proportion of normal weight (BMI 18.5–24.9) and overweight (BMI 25–29.9) participants.

### Change in body age and metabolic syndrome

**Fig 3:**
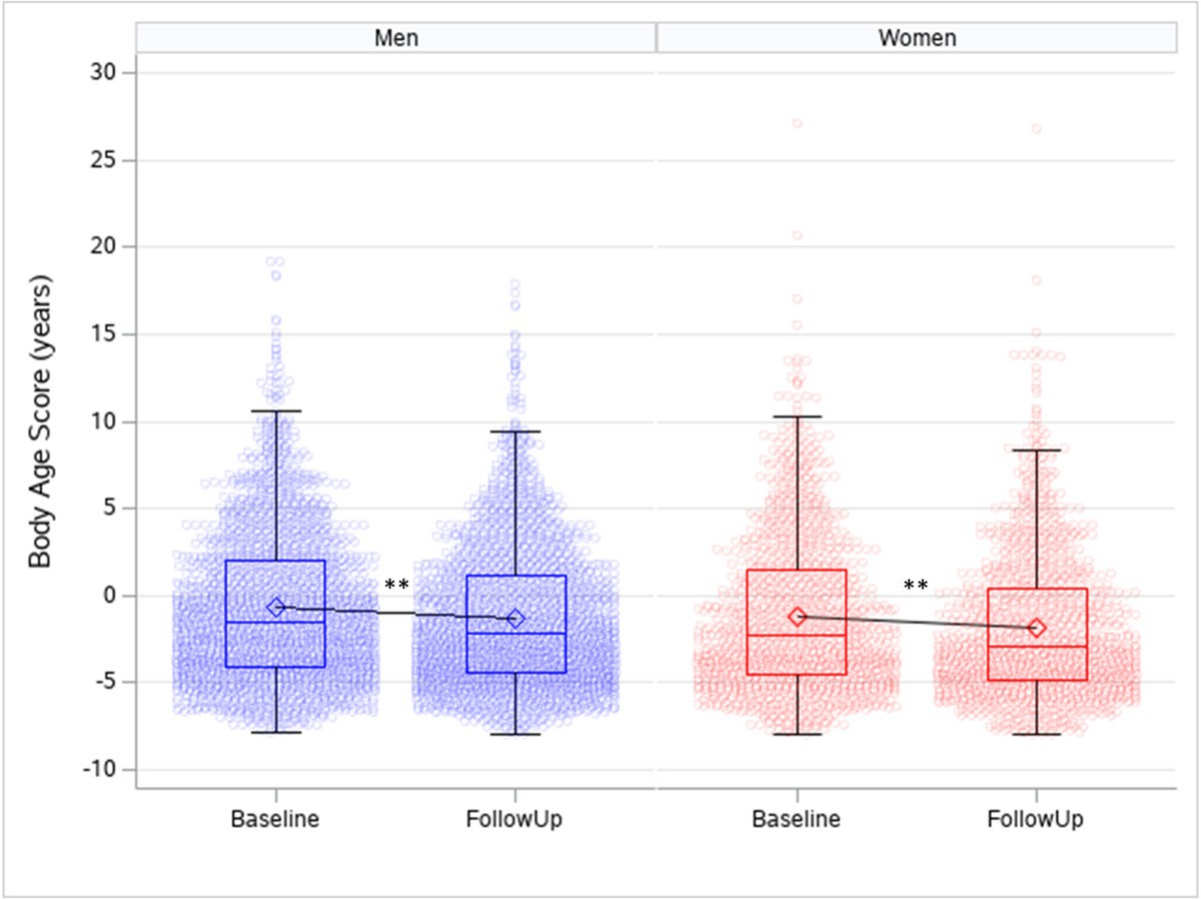
Body age score observed at baseline and follow-up by sex. Men (blue, n = 2251) and women (red, n = 1321). Only those with a body age score comprising all 11 parameters at baseline and follow-up are included in the analysis. P-value using paired t-test. **p<0.*001*.

The median follow up time for 2TP was 1.3 years (IQR 1.0; 2.1 years; min: 0.02 years, max: 5.6 years). Fig 3 shows the body age score at baseline and follow-up for men and women.

**Fig 4:**
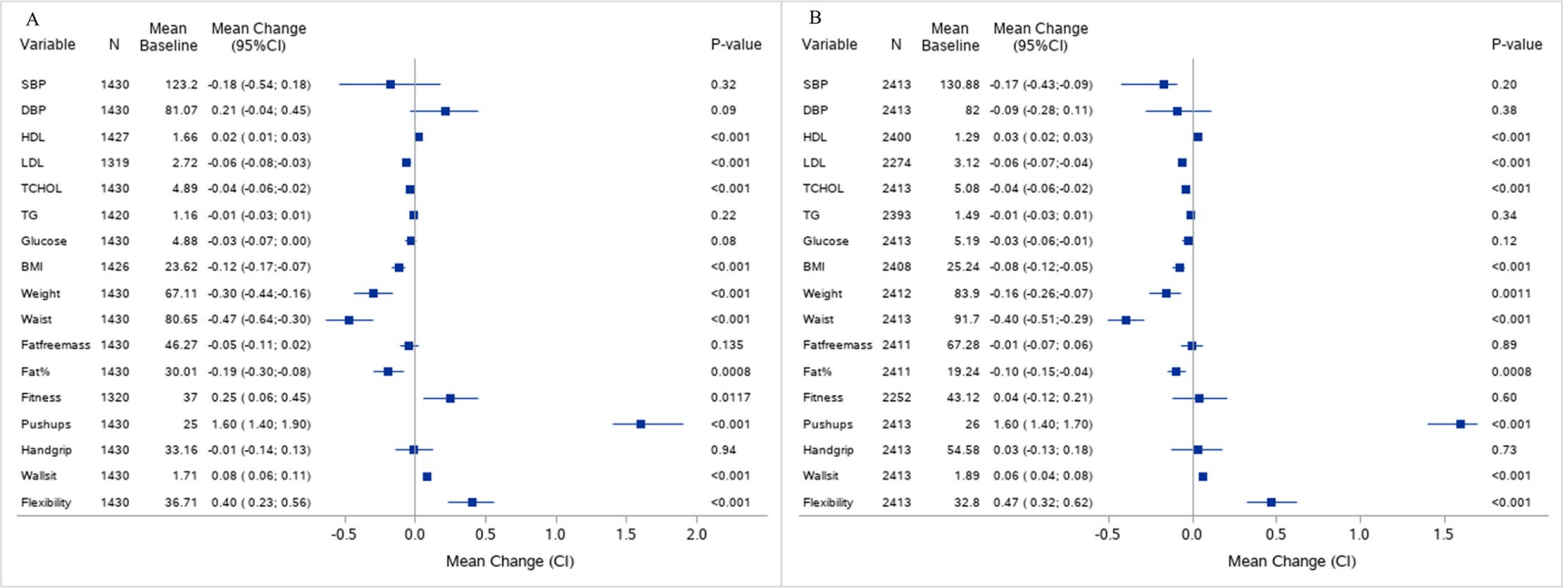
Changes in single variables per year beside the average age development. Baseline values and changes (mean, 95% CI) observed at follow-up adjusted for age at baseline and follow-up time by sex: A = women and B = men. N is the sample size used for calculation of the mean difference. A visualization of the effect size is provided for in the Forest plot; squares representing mean change with 95% confidence intervals. *P value using mixed model adjusted for age at baseline and variation in follow-up time. SBP = systolic blood pressure (mmHg), DBP = diastolic blood pressure (mmHg), HDL = high density lipoprotein (mmol/l), LDL = low density lipoprotein (mmol/l), TCHOL = total cholesterol (mmol/l), TG = triglycerides (mmol/l), Glucose = fasting glucose (mmol/l); BMI = body mass index (kg/m^2^), weight (kg), waist = circumference (cm), fat free mass (kg), fitness level (ml/min/kg), push up = number of, handgrip strength (kg), wall sit time (min) and flexibility = sit and reach test (cm).

Body age improved for both men and women as the mean body age score decreased with -0.6 years (95% CI -0.7; -0.5) and -0.7 years (95% CI -0.8; -0.5), respectively (*p<0*.*001*) (Fig 3). Number of employees with metabolic syndrome had decreased from 646 at baseline to 557 at follow-up (p = 0.005). Fig 4 shows the changes per year beside the average age development in single variables included in the health screening.

After adjusting for age at baseline and variation in follow-up time we found a small but significant positive change in cholesterol for women with and increased HDL (0.02 mmol/l) and decreased LDL (-0.06 mmol/l) concentration. BMI was lower at follow up, together with weight, body fat percent and waist circumference. The same pattern was seen in men. Only women increased their fitness level (0.25 ml/min/kg), but both men and women increased number of push-ups, wall sit time and flexibility indicative of higher physical capacity. No changes in blood pressure or glucose concentration was found in male or female employees.

### Change in smoking habits

At baseline 245 employees smoked in the 2TP group (Table 2). At follow-up, 61% of these had quit/or reduced the number of cigarettes smoked per day (n = 149, no sex differences (*p = 0*.*73*)), quitters representing 42% (n = 103, no sex differences (*p = 0*.*98*)). 21% of smokers (n = 52) increased their cigarette smoking and 18% (n = 43) reported no change. About 0.6% of nonsmokers at baseline had initiated smoking at follow-up (n = 20, no sex differences (*p = 0*.*84*)).

## Discussion

The results of this study show an overall positive effect of using BAI in workplace health promotion. Body age as health-risk estimate improved due to an overall improvement in metabolic risk factors (Fig 4) and an impressive smoking cessation rate of 42%. As visualized in the forest plot in Fig 4 the effect sizes in single metabolic risk factors were small to moderate and the effect on risk of future morbidity could be questioned. This is comparable with effect sizes found in a review on the effect of HRA in workplace health promotion [7] and the reason for the skepticism of using HRA in primary prevention in general [23]. Nevertheless, the changes in metabolic risk factors were associated with a decrease in the number of employees with metabolic syndrome, commonly used in the clinic to assess individuals with high risk of future cardiovascular disease and type 2 diabetes [24, 25]. Furthermore, small effect sizes have been found to independently affect the risk of non-communicable diseases. Data from the Framingham Offspring Study suggest that 0.055 mmol/L increase in HDL relates to a 2–3% decrease in risk of CVD [26], which is somewhat similar to the effect sizes seen in our study.

Reducing the proportion of employees who smoke, substantially reduce absenteeism from work as well as the duration of absenteeism [27]. Our finding that 42% had quit smoking is high compared to previous results from workplace health promotion interventions showing cessation rates of 20.3% and 8.1% [7, 28] but lower compared to workplace interventions solely focusing on smoking prevention (53.2%) [29]. The unique combination of MI and the awareness of the influence smoking have on body age might be important determinants when changing smoking behavior. Studies on anti-smoking interventions have shown that MI outperforms traditional advice [30, 31]. On the other hand, the documented effect of body age as a motivational tool is sparse. A cluster randomized study (n = 121) of healthy workers found that body age assessment did not increase encouragement and motivation for changes in health behavior [11]. In contrast a randomized study on heart patients (n = 660), found that patients aware of the net-value (change in body age) were more likely to choose certain health-risk behavior to change if the change resulted in a high reduction in body age [10]. Our finding on smoking cessation could have been affected by secular trends and not the intervention per se. Following the new Danish law against smoking at public- and workplaces in 2007, 320.00 Danes quit smoking. However, from 2011 to 2016 the amount of smokers was stable at 21–23% comprising almost the entire sample period [32].

Comparison of 1- and 2TP at baseline suggest that 1TP are less healthy (Table 2). Lower participation rate among the unhealthiest employees is a known issue in workplace health promotion [33]. Fig 2 also indicate that employees with the highest body age score are less likely to participate at follow-up especially within the oldest part of the study population. However, we acknowledge that the highly significant differences between groups in BMI, mean blood pressure, waist circumference and body fat percentage (Table 2) could be ascribed to the large sample size, and it can be questioned whether the differences between the groups have clinical relevance thus, if the two groups differ from a health risk perspective. On the other hand, smoking increase the risk of premature death mediated through cardiovascular- and lung diseases [34]. Thus, a higher prevalence of smokers in the 1TP group will increase their risk of future lifestyle related diseases. Also, as smoking has great impact on body age we speculate that reluctance to change smoking behavior influenced the motivation to attend at follow-up. Another strong predictor for cardiovascular disease and premature mortality is fitness level [35]. We found a median difference between the groups of 3 ml/kg/min (Table 2). Studies have shown that an increase of 1MET (approximately 3.5 ml/kg/min) in fitness level was associated with 21% lower risk of future CVD [36] and that a 1 ml/kg/min increase in fitness level was equivalent to a CVD risk reduction of 10% and 9% in women and men respectively [37]. Collectively, these results indicate that 1TP have higher risk of future lifestyle related diseases despite the close values of medians at baseline between the groups.

Other limitations include that body age as a health risk-estimate has not been tested for reliability, although it includes separate measurements that have been used and tested. Wall sit-hold and push up are susceptible for bias due to a learning effect, thus the improvement in body age seen at follow-up could partly be due to this. The study design does not allow for causal conclusions and we do not know if other health promotion activities have been conducted by the companies within the follow-up time. No information on drug use was given, and some of the changes we found could be assigned to drug initiation. Smoking habits were self-reported which might inflate the result regarding reduced CPD. Finally, it is worth noting that factors like alcohol intake, education level, socioeconomic status and marital status could confound the interpretation of change in smoking habits and metabolic syndrome. The strengths of this study are the performance in a real-life setting, in a large sample of the Danish workforce using a novel approach to worksite health promotion. This improves the generalizability that formal RCT-like designs struggles with. Our study shows that in a real-life setting 70% of the invited employees wanted to participate in at least 1 health risk assessment. High participation rate is a crucial part of successful health promotion and the result implies that body age assessments attracts employees across work fields and across a wide health profile spectrum (`2). In comparison the Inter99 study, a large Danish population-based randomized longitudinal study, using individual health risk assessment plus individual and group-based counselling as intervention, had a baseline participation rate of 52.4% [38]. The Inter99 study was conducted in the participants’ spare time whereas our study was conducted during working hours, which have been shown to be important determinants for participation rate [39]. However, we are aware that the participation rate observed at baseline attenuates through follow-up. Effort to reduce lost to follow-up is a recurring issue in health promotion in general, why future research on this matter should be highly prioritized.

## Conclusion

In this study we investigated BAI as workplace health promotion in a large representative sample of the Danish workforce. The effectiveness on single metabolic risk factors were small and due to the study design difficult to translate into effect on risk of future disease. Even so, using BAI including MI was associated with a decrease in the proportion of employees with metabolic syndrome and a surprisingly high smoking cessation rate of 42%. This could indicate that body age as health-risk estimate makes it easy to understand to what extent health behavior affects vigor and risk of disease and thus has potential as motivational tool in health promotion. This study demonstrate that BAI including MI is feasible on a large scale as workplace health promotion, but further research should be aimed towards a) validating body age towards morbidity and b) comparing BAI with standard HRA in workplace health promotion and c) qualitatively assessing body age as motivational tool in order to recommend BAI as a tool in health promotion.

## Supporting information

S1 Checklist STROBE statement—Checklist of items that should be included in reports of observational studies. (DOC)
