# Supplementary material for: The effectiveness of body age-based intervention in workplace health promotion: Results of a cohort study on 9851 Danish employees
Source: PLoS One. 2020 Sep 17;15(9):e0239337. doi: 10.1371/journal.pone.0239337 (PMC7498070; doi:10.1371/journal.pone.0239337)
Supplement: S1 Checklist — (DOC) [file pone.0239337.s001.doc]

STROBE Statement—checklist of items that should be included in reports of observational studies

|  | Item No | Recommendation |
| --- | --- | --- |
| **Title and abstract** | 1 | (*a*) Indicate the study’s design with a commonly used term in the title or the abstract **line 1-3 page 1** |
|  |  | (*b*) Provide in the abstract an informative and balanced summary of what was done and what was found. **Line 16-34 page 2** |
| Introduction | | |
| Background/rationale | 2 | Explain the scientific background and rationale for the investigation being reported. **Line 36-57 page 2-3** |
| Objectives | 3 | State specific objectives, including any prespecified hypotheses. **Line 58-62 page 3.** |
| Methods | | |
| Study design | 4 | Present key elements of study design early in the paper. **Line 66 page 4.** |
| Setting | 5 | Describe the setting, locations, and relevant dates, including periods of recruitment, exposure, follow-up, and data collection. **Line 66-68 page 4.** |
| Participants | 6 | (*a*) *Cohort study*—Give the eligibility criteria, and the sources and methods of selection of participants. Describe methods of follow-up. **Line 67-93 page 4-5**  *Case-control study*—Give the eligibility criteria, and the sources and methods of case ascertainment and control selection. Give the rationale for the choice of cases and controls  *Cross-sectional study*—Give the eligibility criteria, and the sources and methods of selection of participants |
|  |  | (*b*) *Cohort study*—For matched studies, give matching criteria and number of exposed and unexposed. **N.a.**  *Case-control study*—For matched studies, give matching criteria and the number of controls per case |
| Variables | 7 | Clearly define all outcomes, exposures, predictors, potential confounders, and effect modifiers. Give diagnostic criteria, if applicable. **Line 59-62 page 3** **and line 162-163 page 8** |
| Data sources/ measurement | 8* | For each variable of interest, give sources of data and details of methods of assessment (measurement). Describe comparability of assessment methods if there is more than one group. **Line 108-153 page 5-7.** |
| Bias | 9 | Describe any efforts to address potential sources of bias. **Line 98-101 page 5** |
| Study size | 10 | Explain how the study size was arrived at. **Line 95-101 page 5** |
| Quantitative variables | 11 | Explain how quantitative variables were handled in the analyses. If applicable, describe which groupings were chosen and why. **Line 95-98 page 5 and 156-161 page 8** |
| Statistical methods | 12 | (*a*) Describe all statistical methods, including those used to control for confounding. **Line 154-163 page 8** |
|  |  | (*b*) Describe any methods used to examine subgroups and interactions. **N.a.** |
|  |  | (*c*) Explain how missing data were addressed. **Line 157-158 page 8** |
|  |  | (*d*) *Cohort study*—If applicable, explain how loss to follow-up was addressed. **N.a.**  *Case-control study*—If applicable, explain how matching of cases and controls was addressed  *Cross-sectional study*—If applicable, describe analytical methods taking account of sampling strategy |
|  |  | (*e*) Describe any sensitivity analyses. **N.a.** |

| Results | | |
| --- | --- | --- |
| Participants | 13* | (a) Report numbers of individuals at each stage of study—eg numbers potentially eligible, examined for eligibility, confirmed eligible, included in the study, completing follow-up, and analysed. **Line 165 page 8** |
|  |  | (b) Give reasons for non-participation at each stage **%** |
|  |  | (c) Consider use of a flow diagram **Fig 1** |
| Descriptive data | 14* | (a) Give characteristics of study participants (eg demographic, clinical, social) and information on exposures and potential confounders. **Table 2** |
|  |  | (b) Indicate number of participants with missing data for each variable of interest. **Figur 4** |
|  |  | (c) *Cohort study*—Summarise follow-up time (eg, average and total amount). **Line 196 page 10** |
| Outcome data | 15* | *Cohort study*—Report numbers of outcome events or summary measures over time. **Line 195-226 page 10-11** |
|  |  | *Case-control study—*Report numbers in each exposure category, or summary measures of exposure |
|  |  | *Cross-sectional study—*Report numbers of outcome events or summary measures |
| Main results | 16 | (*a*) Give unadjusted estimates and, if applicable, confounder-adjusted estimates and their precision (eg, 95% confidence interval). Make clear which confounders were adjusted for and why they were included. **Table 2 and Figure 4** |
|  |  | (*b*) Report category boundaries when continuous variables were categorized. **Line 192-194 page 10** |
|  |  | (*c*) If relevant, consider translating estimates of relative risk into absolute risk for a meaningful time period.**%** |
| Other analyses | 17 | Report other analyses done—eg analyses of subgroups and interactions, and sensitivity analyses. **%** |
| Discussion | | |
| Key results | 18 | Summarise key results with reference to study objectives. **Line 228-230 page 11** |
| Limitations | 19 | Discuss limitations of the study, taking into account sources of potential bias or imprecision. Discuss both direction and magnitude of any potential bias. **Line 252-267 page 12** |
| Interpretation | 20 | Give a cautious overall interpretation of results considering objectives, limitations, multiplicity of analyses, results from similar studies, and other relevant evidence. **Line 230-277 page 12-13** |
| Generalisability | 21 | Discuss the generalisability (external validity) of the study results. **Line 283-285 page 13** |
| Other information | | |
| Funding | 22 | Give the source of funding and the role of the funders for the present study and, if applicable, for the original study on which the present article is based. **Funding is described as part of submission procedure.** |

*Give information separately for cases and controls in case-control studies and, if applicable, for exposed and unexposed groups in cohort and cross-sectional studies.

**Note:** An Explanation and Elaboration article discusses each checklist item and gives methodological background and published examples of transparent reporting. The STROBE checklist is best used in conjunction with this article (freely available on the Web sites of PLoS Medicine at http://www.plosmedicine.org/, Annals of Internal Medicine at http://www.annals.org/, and Epidemiology at http://www.epidem.com/). Information on the STROBE Initiative is available at www.strobe-statement.org.
